# Assessment of GC-MS in Detecting Changes in the Levels of Metabolites Using a Spike-in Experiment in Human Plasma

**DOI:** 10.4172/2153-0769.1000175

**Published:** 2016-06-03

**Authors:** Rency S. Varghese, Cristina Di Poto, Chao Tu, Mohammad R. Nezami Ranjbar, Yue Luo, Jiwon Choi, Mahlet G. Tadesse, Habtom W. Ressom

**Affiliations:** 1 Department of Oncology, Lombardi Comprehensive Cancer Center, Georgetown University Medical Center, Washington, DC, USA; 2 Department of Mathematics and Statistics, Georgetown University, Washington, DC, USA

**Keywords:** Metabolomics, GC-MS, Spike-in experiment, Internal standards, Latin square design

## Abstract

Gas Chromatography coupled with Mass Spectrometry (GC-MS) has been broadly used for the detection of changes in metabolite levels in complex samples. Internal Standards (IS) spiked into a complex background at different concentrations help assess the capability of GC-MS in detecting changes in metabolite levels. This study uses a Latin square design to evaluate the ability of GC-MS in full scan and Single Ion Monitoring (SIM) modes to detect changes among IS spiked into human plasma samples at varying concentrations. Statistical analysis of the data demonstrates the potential of GC-MS to detect true differences over a wide range of concentration levels.

## Introduction

Metabolomics studies small molecules (molecular weight < 1800 Da) that define the metabolic status of a biological system. This technique provides a simultaneous assessment of numerous metabolites that can help the characterization of phenotypic profiles and also the quantification of individual metabolites. Untargeted and targeted metabolomic methods are used to evaluate the changes in levels of metabolites between biologically distinct groups. In untargeted metabolomics, spectral features representing known and unknown metabolites are processed chemo metrically to select metabolites with significant differences between the biological groups. In targeted metabolomics, compounds are first identified prior to quantification for difference detection. Mass spectrometry (MS) has been extensively used for both untargeted and targeted metabolomic studies because of its accuracy, sensitivity, and coverage [1–3]. Chromatography is often coupled to mass spectrometry to achieve better separation of multiple compounds present in a complex matrix before their ionization. Both gas chromatography (GC) and liquid chromatography (LC) have been used in metabolomics studies to increase the metabolome coverage. In Gas Chromatography coupled to Mass Spectrometry (GC-MS), after separation by chromatography of vaporized compounds, a mass spectrometer is used to identify and quantify small molecule metabolites. GC-MS has been widely used in forensic science, environmental analysis, drug detection and to perform analysis specifically for the detection and identification of substances. Most recently, with the emerging metabolomics discipline in biofluid analysis, GC-MS is being used along with other analytical platforms (LC-MS and NMR) to achieve higher metabolome coverage by overcoming the challenge of separating and identifying metabolites with different polarity characteristics. One of the motivations for conducting metabolomic studies is the potential to diagnose disease status based on metabolites present in non-invasive bio specimens such as blood or urine. There is a growing need to identify biomarkers as measureable indicators, most importantly when trying to predict early stages of the disease in asymptomatic subjects [4–6]. An advantage of GC-MS over LC-MS for metabolomics analysis is the availability of commercial spectral libraries and structure databases that can be used for metabolite identification.

The reliability and utility of comparative metabolite profiling studies is critically dependent on an accurate and rigorous assessment of the quantitative changes [7]. An internal standard is a compound added to sample at a known concentration. It is typically similar, but not identical to the chemical species of interest in the sample, so that it can be identified during the analysis of the mass spectral data consisting of signals corresponding to the standards as well as those from the sample. We refer to adding known quantities of analyte (s) of interest (e.g., internal standards) into a sample as “spike-in”. The use of well distinguishable internal standards, spiked into a complex background, at different concentration levels allows us to assess the ability of GC-MS to accurately detect true changes in metabolite levels.

In a previous study [8], we conducted a spike-in experiment to evaluate computational methods to detect changes in protein expression levels measured by Liquid Chromatography combined with Mass Spectrometry (LC-MS). Two proteomic datasets were generated with presence or absence of internal standards spiked in human serum samples. Various software tools were evaluated in their ability to identify the true differences between the two datasets.

In this study, we used five isotopically labeled internal standards to create five mixtures to be spiked into the complex sample background of human plasma. Each mixture contained the five internal standards at varying concentrations following a 5-by-5 Latin square array [9,10]. Prior to metabolite extraction, the five mixtures of IS were spiked into five aliquots of plasma samples derived from five healthy individuals. The samples were then analyzed using three GC-MS platforms, (i) An Agilent GC coupled to a LECO TOF mass spectrometer (GC-TOF-MS); (ii) An Agilent GC coupled with an Agilent single quadrupole mass spectrometer (GC-qMS) operated at full scan; and (iii) The GC-qMS operated in Single Ion Monitoring Mode (GC-SIM-MS). When generating data in full scan mode, a wide range of masses was acquired (scan range m/z 50–600). In SIM mode, instead, the mass spectrometer was set to monitor specific ion fragments gaining higher sensitivity due to the ability of the mass spectrometer to dwell for a longer time. The data acquired from these platforms were used to detect known differences among internal standards spiked into the background at varying concentrations. A set of plasma endogenous metabolites was also monitored for quality assessment.

The results of this study demonstrate the capability of GC-MS, operating both in full scan and SIM modes, to detect internal standards spiked into plasma samples. By assessing the quantitative changes among five different mixtures of IS and their reproducibility among five healthy control subjects, it is shown that GC-MS can detect changes in the levels of isotopically labeled internal standards spiked-in human plasma samples at varying concentrations.

## Methods

### Materials

Deuterium labeled internal standards were purchased from CDN isotopes (Pointe-Claire, QC, Canada). These include L-phenyl-d5-alanine-2,3,3,-d3 (D-1241), L-glutamic-2,3,3,4,4-d5 acid (D-899), L-alanine-2,3,3,3-d4 (D-1488). Glycine–d5 (175833), myristic acid–d27 (366889), the fatty acid methyl ester standards, C8 (260673), C9 (245895), C10 (299030), C12 (234591), C14 (P5177), C16 (P5177), C18 (S5376), C20 (10941), C22 (11940), C24 (87115), C26 (H6389), C28 (74701), methoxyamine hydrochloride (226904) and pyridine (360570) were purchased from Sigma Aldrich (St. Louis, MO, USA). C30 (T0812) was purchased from TCI chemicals (Portland, OR USA-T0812). MSTFA (TS-48910) was purchased from Thermo Scientific (Waltham, MA, USA). HPLC grade 2-propanol, acetonitrile and water were used for metabolites extraction. Helium was purchased from Robert Oxygen (Rockville, MD, USA).

### Sample collection

Blood samples were obtained from five healthy individuals recruited at Georgetown University who provided informed consent to the study as approved by the University Institutional Review Board. Through peripheral venipuncture, blood was drawn into 10 mL BD Vacutainer sterile vacuum tubes in the presence of EDTA anticoagulant. The blood was immediately centrifuged at 1000 g for 10 min at room temperature. The plasma supernatant was carefully collected and centrifuged at 2500 g for 10 min at room temperature. After aliquoting, plasma was kept frozen at −80°C until use.

### Experimental design and sample preparation

**Figure 1:**
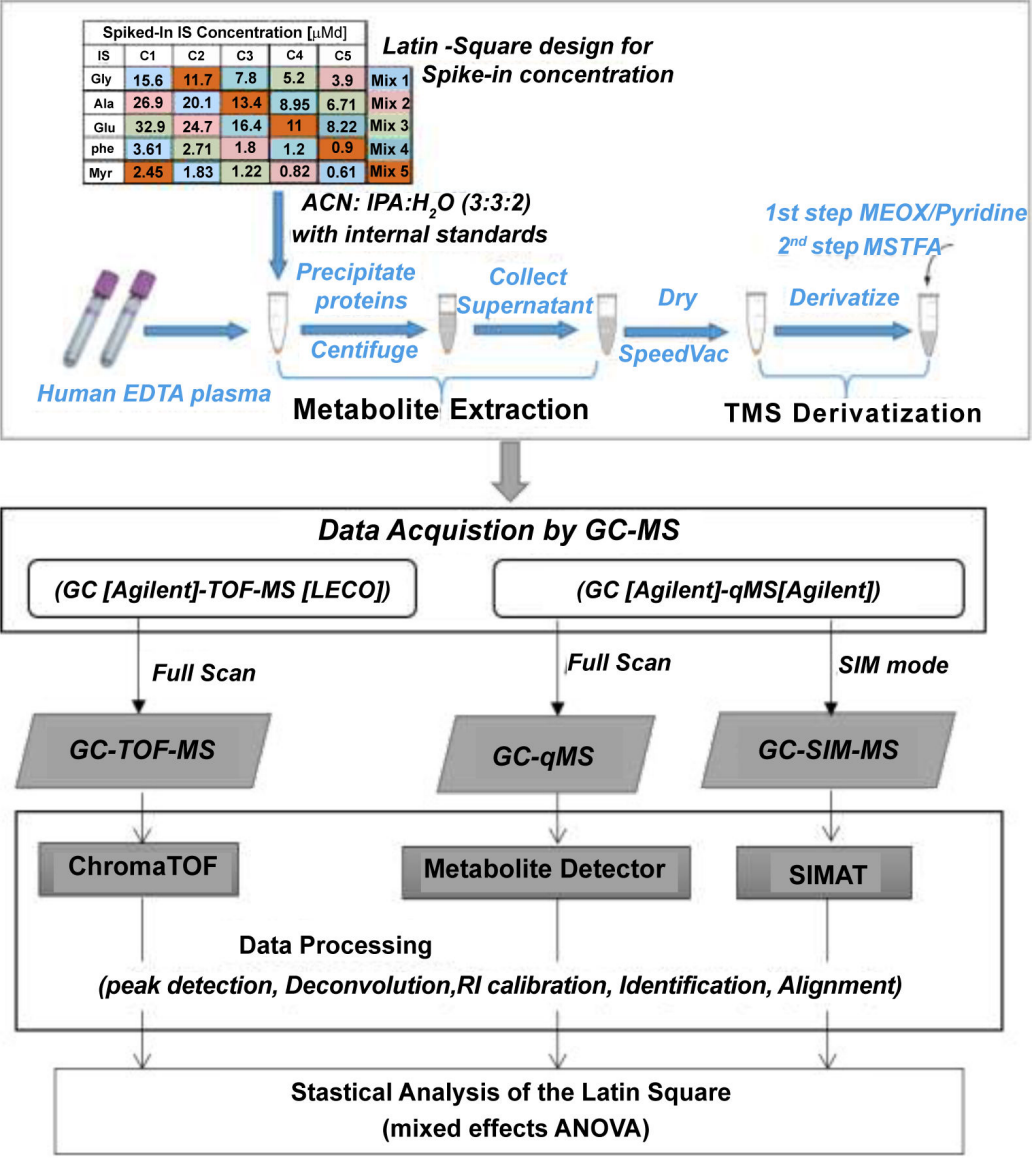
Overview of the spike-in experiment.

**Figure 2:**
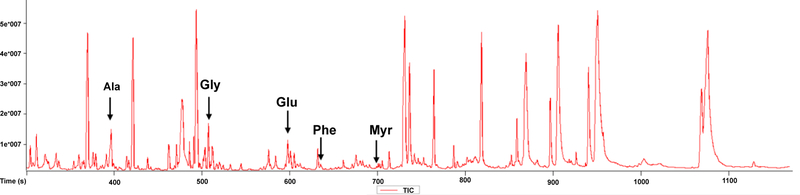
Total Ion Chromatogram (TIC) for one of the plasma runs of the spike-in experiment.

**Figure 3:**
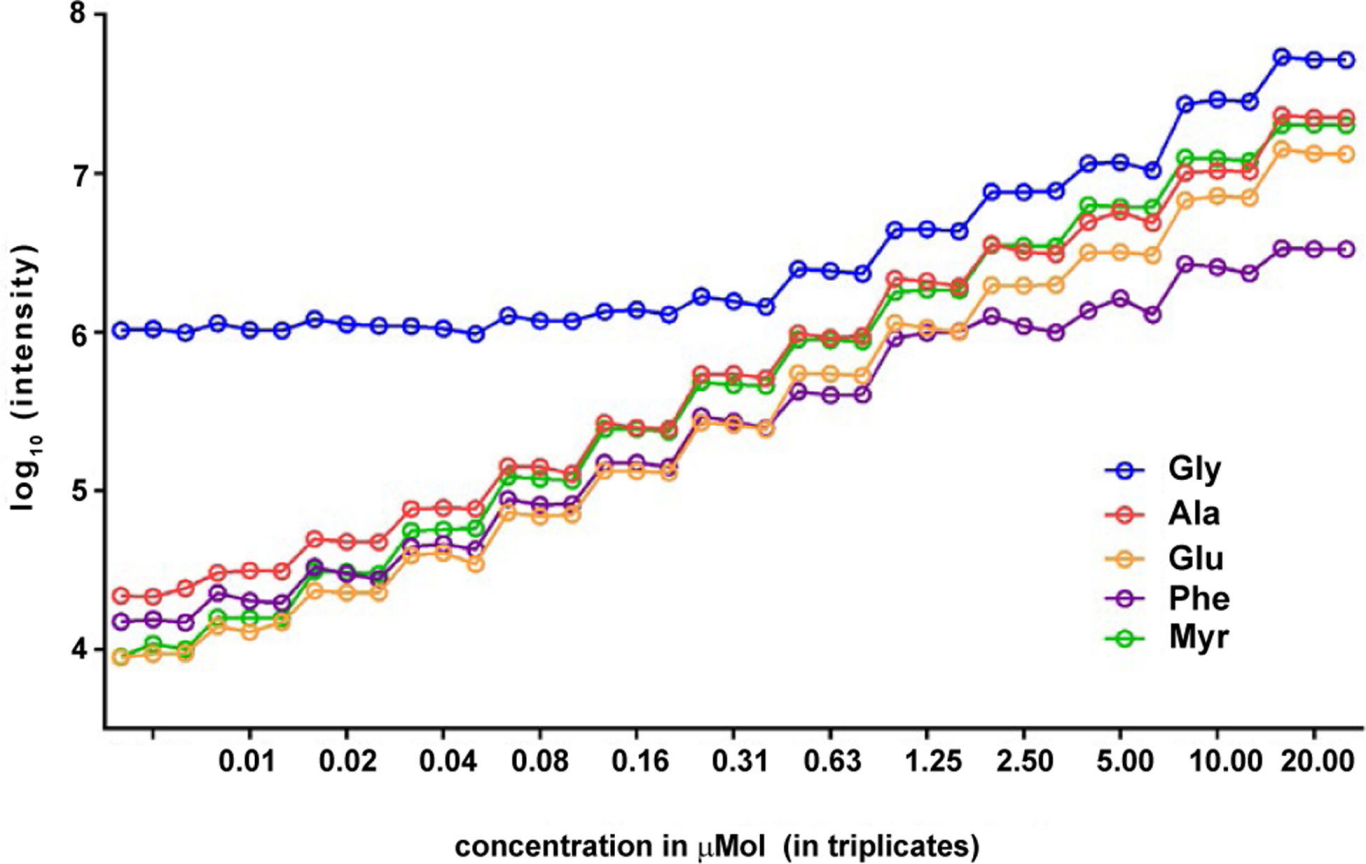
Ion intensity measurements of five IS at 13 concentrations.

Figure 1 illustrates the experimental design of the spike-in study. Each spike-in mixture (Mix1–Mix5) contained five internal standards (Glycine–d5 (Gly), Glutamic acid-2,3,3,4,4-d5 (Glu), Alanine-2,3,3,3-d4 (Ala), Phenylalanine-phenyl-d5–2,3,3,-d3 (Phe), and Myristic acid d27 (Myr) at varying concentration levels was dissolved in 1 mL of working solution composed of acetonitrile, isopropanol, and water (3:3:2). To evaluate the effect of the matrix, we looked into the Total Ion Chromatogram (TIC) for one of the plasma runs of the spike-in experiment (Figure 2). As shown in the figure, the peaks that correspond to the peak intensities of the internal standards are smaller than most of the high intensity peaks corresponding to the plasma metabolites. For each IS, we specified a concentration level (C_0_) on the basis of ion intensity measurements detected by GC-MS with S/N ≥ 10 for 13 different concentration levels of each IS ranging from 0.0049 to 20 μM. As shown in Figure 3, unlike the other four IS, the intensities of Gly did not follow the concentration trend at the low concentration levels. Based on this, we selected the spike-in concentration for Gly from the high concentration range. The final spike-in concentration levels for the five IS used in the spike-in experiment ranged from 0.6–32.8 μM.

To evaluate the ability to detect changes in metabolite levels over a range of concentrations, we generated GC-MS data from different mixtures following a Latin square array design [10] as illustrated in Table 1. C_0_ was selected for each IS based on the initial experiment, and C_1_ to C_5_ were determined by multiplying C_0_ by 0.5, 0.67, 1, 1.5, and 2 fold, respectively. We spiked-in five mixtures of isotopically labeled standards to human plasma collected from five healthy volunteers. Plasma metabolites were extracted after adding each mixture to 30 μL of plasma. After vortexing, samples were centrifuged at 14,500 g for 15 minutes at room temperature. The supernatant was then divided into two, 460 μL each, for analysis by GC-TOF-MS and GC-qMS systems. Each supernatant was then concentrated to dryness in speedvac. The dried samples were kept at −20°C until derivatization prior to analysis by GC-MS. Our sample preparation method including derivatization can be found in reference [11]. Samples were acquired by GC-TOFMS, GC-qMS, and GC-SIM-MS. For quality assessment, we included the following three approaches in the experimental design: (i) A Retention Index (RI) standard mixture at the beginning and at the end of the GC-MS data acquisition. The standard was prepared by mixing a series of Fatty Acid Methyl Esters (FAMEs). Specifically, FAMEs, including C8, C9, C10, C12, C14, C16, C18, C20, C22, C24, C26, C28 and C30 linear chain length were dissolved in chloroform at a concentrations of 0.8 mg/mL (C8–C16) and 0.4 mg/ml (C18–C30). 100 μL of each FAME standard was mixed together and 1.2 mL of chloroform was added for a final volume of 2.5 mL (FAME1). A ratio of 1:19 FAME1/Hexane was mixed prior to analysis by GC-MS. (ii) Blank samples were prepared by adding the derivatization agent to an empty tube and following the same steps as the other spike-in samples. (iii) A pooled QC sample, obtained by taking an equal volume from each prepared sample, was run multiple times at the beginning of the queue for column conditioning.

### GC-MS data acquisition and preprocessing

The metabolites extracted from the plasma samples spiked with IS were analyzed by GC-TOFMS, GC-qMS in full scan mode, and GC-qMS in SIM mode. The three datasets acquired by these platforms are denoted in this paper as GC-TOF-MS, GC-qMS, and GC-SIM-MS, respectively. A detailed description of our GC and MS methods for both platforms can be found in [11]. The data generated by GC-TOFMS and GC-qMS were converted to the standard netCDF format. ChromaT of was used to preprocess the data generated using GC-TOF-MS. Metabolite Detector [12] and SIMAT [13] was used to preprocess GC-qMS and GC-SIM-MS. The data acquired by GC-TOF-MS and GC-qMS were preprocessed by utilizing Retention Indices (RI). In Metabolite Detector, RI values are calculated for every detected analyte based on RI and RT values of FAMEs mixture run at the beginning of the analysis. These values are used to correct the alignment of metabolites across multiple run. ChromaT of allows the calculation of analytes’ RI though a calibration curve. These values were used for narrowing the library search results, thereby reducing the number of false putative identifications. Putative identifications were determined based on spectral matching using the Fiehn library.

For GC-SIM-MS, the RTs of a subset of the targets with very high similarity scores were detected and compared against those in the Fiehn library to estimate the difference between expected and observed elution times. Following that, we used SIMAT [13] to read the CDF files and extract the EIC guided by the estimated RT from the previous step. The algorithm uses an RT window centered at the expected elution time of the analyte of interest and searches the neighborhood area for all detected peaks at monitored masses. The quantifier fragment is used to perform the search and all qualifiers peaks are found based on the location of the quantifier peak. Also, smoothing of the EICs and baseline correction are performed before peak detection. Then, the peak width and the Area Under the Curve (AUC) of each EIC are calculated. Finally, a similarity score is calculated based on the expected SIM spectra from the library to evaluate the goodness of identification. Specifically, a mixed measure is used to calculate the similarity score based on weighted dot product and average pairwise ratios between fragments. All EICs are checked by visual inspection to avoid identification errors.

### Latin square design

In combinatorics and in experimental design, a Latin square is an n × n array filled with n different symbols, each occurring exactly once in each row and exactly once in each column. The Latin square design is typically used for a situation in which there are two extraneous sources of variation. It allows for two blocking factors in controlling two sources of variability. If the rows and columns of a square correspond to the levels of the two extraneous variables, then in a Latin square each treatment appears exactly once in each row and column [9]. The data we acquired from both instruments were preprocessed and normalized using the intensities measured at defined concentration C_0_, for each internal standard and rearranged to follow the 5 × 5 Latin square experimental design (Table 1), with five rows and five columns, where each row represents an internal standard and each column is a mixture factor. The resulting 25 cells contain one of the five concentrations and each concentration can only occur once in each row and column. Deviations caused by two independent factors have been cancelled out of calculation of the effect of interest, leaving an evaluation of the principal effect unaffected by the nuisance effects. The model of the Latin Square design is assumed additive, that is, no interaction between factors (internal standards, mixture) and the treatment (concentrations). However, the degree of freedom for error is often too small because of the small size of the square. Thus, we considered replicating the Latin Square five times with five biological replicates for a total of 125 observations. The internal standards and mixtures are kept the same and the same experimental method is used in each replication.

### Statistical analysis

We consider the internal standards, mixture and concentration as fixed effects while the subject replicates are viewed as random effect. Estimation of the main effects and the variance terms from the replicated Latin Square design can be done using a mixed effects Analysis of Variance (ANOVA) model. The statistical programming software R was used to fit the model:

$${\text{Y}}_{\text{hijk}}=\text{μ}+{\text{η}}_{\text{η}}+{\text{θ}}_{\text{i}}+{\text{φ}}_{\text{k}}+{\text{ρ}}_{\text{j}}+{\text{ε}}_{\text{hijk}},\left(\text{h},\text{i},\text{j}=1,\ldots 5;\text{k}=\left({\text{C}}_{1},{\text{C}}_{2},{\text{C}}_{3},{\text{C}}_{4},{\text{C}}_{5}\right)\right)\;\text{​}{\text{ρ}}_{\text{j}}\sim \text{N}\left(0,{\text{τ}}^{2}\right){\text{ε}}_{\text{hijk}}\sim \text{N}\left(0,{\text{σ}}^{2}\right)$$

where μ is the overall mean, ƞ is the row block effect, Ɵ is the column effect, φ is the treatment effect and ρ is the random replicate effect from a population with mean zero and variance τ^2^.

## Results and Discussion

**Figure 4:**
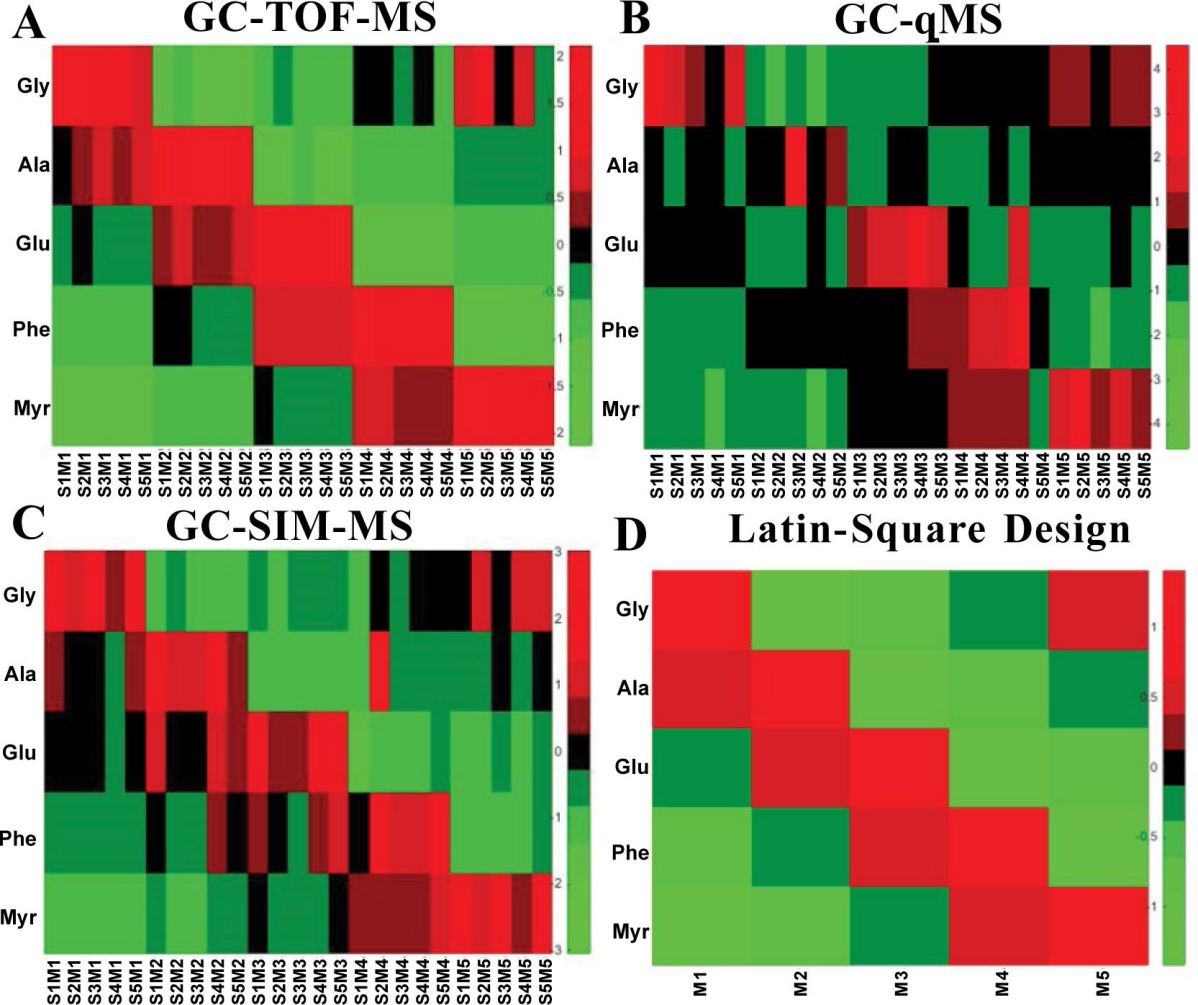
Heatmaps of the normalized intensities for the five IS acquired across all samples and mixtures using (A) GC-TOF-MS, (B) GC-qMS, (C) GC-SIM-MS, and (D) the actual concentrations/design.

**Figure 5:**
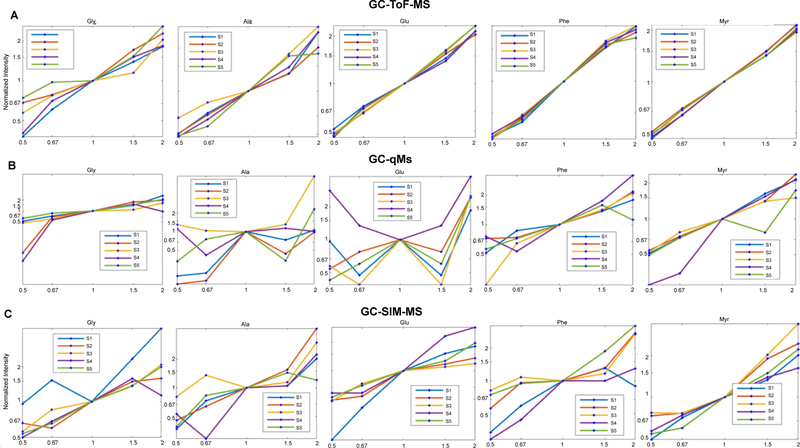
Fold-change measured at 5 concentrations (C1–C5) for each internal standard for data acquired using (A) GC-TOF-MS, (B) GC-qMS, and (C) GC-SIM-MS.

**Figure 6:**
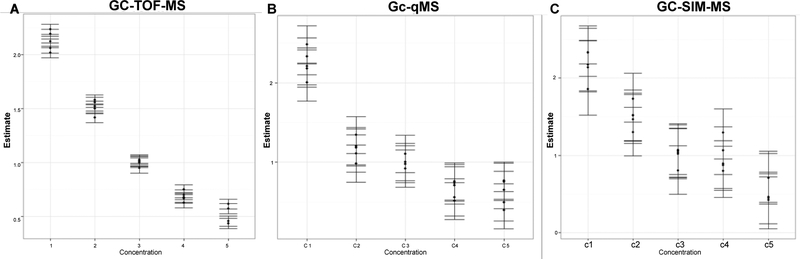
Conditional means of concentration for each level with standard error in five replicates for (A) GC-TOF-MS, (B) GC-qMS, and (C) GC-SIM-MS.

To understand the behavior of the IS among the 5 mixtures and 5 biological replicates, we extracted the main effects and the relevant variance from the replicated latin square design by applying the mixed effect Analysis of Variance (ANOVA) model. Table 2 shows the p-values obtained from the ANOVA model for the data generated by GC-TOF-MS, GC-qMS, and GC-SIM-MS. As illustrated in the table, significant differences were observed among the five concentrations adjusting for the effects of mixtures and IS. For each IS, we were able to measure the change in concentration spiked into the mixtures. This change in levels is also exemplified using heatmaps for all three datasets and the design matrix (Figure 4). Figure 5 depicts the fold changes measured at 5 Concentrations (C1–C5) for each internal standard across all subjects. As can be gathered from the figure, for each internal standard we were able to measure the change in concentration spiked into the mixtures. In addition, we performed a t-test between the peak intensities at different concentrations for each IS. Using the data acquired by GC-TOF-MS, we observed statically significant changes in concentration levels for all the internal standards. The data acquired by GC-SIM-MS also detected the changes in concentration levels, but they were for the most part marginally significant. Through the data acquired by GC-qMS, we were able to detect significant changes for Myr and Gly at all concentrations. However, the change in concentration levels for Phe was not significant in the low concentration range (0.5 and 0.67). On the other hand, although GC-qMS detected Ala and Glu at each concentration, the changes in concentration levels were not statistically significant. The changes in concentration level for Myr were statistically significant in data acquired by all three platforms. The main effect of concentration is illustrated in Figure 6 using the conditional mean and standard error of concentration for each level. As shown in the figure, differences exist among the concentration levels when controlling the other effects. Thus, the replicated Latin Square design is applicable to test the main effect of varying concentrations in this experiment.

Although we did not anticipate any changes in the intensity of the endogenous metabolites since the only compounds varying in concentrations were the spiked-in internal standards, we evaluated the behavior of plasma metabolites in the data acquired by GC-TOF-MS and GC-qMS by calculating their Coefficients of Variation (CVs) across runs within the same subject and across the five subjects. As expected, we observed smaller CVs, ranging between 1–3% compared to the CVs calculated for the IS (Table 3). A high degree of linearity is also observed for signal intensity versus concentration of the endogenous metabolites. This linearity in signal response, together with a high degree of reproducibility reflected in low median and mean CVs demonstrates the potential of GC-MS instruments to detect changes in metabolite levels.

## Conclusion

In our study, we demonstrated the ability of three platforms/instrument methods (GC-TOF-MS, GC-qMS, and GC-SIM-MS) to capture significant changes in the levels of internal standards spiked in human plasma over a pre-defined range of concentrations. The changes in concentration levels measured by the GC-TOF-MS are more consistent and statistically significant for all the internal standards, compared to those measured by the GC-qMS and GC-SIM-MS. The use of Latin square design for situations where there is more than one source of variation provided greater power to evaluate the differences in the spiked-in concentrations. It also allowed us to conduct the spike-in experiment with small number of runs. This spike-in experiment demonstrates the potential application of GC-MS instruments in detecting differences in metabolite levels over specified concentration levels. Future work will focus on determining dynamic range, particularly, the ability to detect metabolites with lower concentrations.

## Figures and Tables

**Table 1:** Latin square design.

Spiked-ln IS Concentration (μMol)							
IS	C1=0.5C_0_	C2=0.67C_0_	C3=1C_0_	C4=1.5C_0_	C5=2C_0_		
**Gly**	3.9	5.2	7.8	11.7	15.61		Mix 1
**Ala**	6.71	8.95	13.42	20.14	26.85		Mix 2
**Glu**	8.22	10.95	16.43	24.65	32.86		Mix 3
**Phe**	0.9	1.2	1.8	2.71	3.61		Mix 4
**Myr**	0.61	0.82	1.22	1.83	2.45		Mix 5

**Table 2:** P-values of mixed effects ANOVA models for data acquired by GC-TOF-MS, GC-qMS, and GC-SIM-MS in Latin-square design.

GC Instrument & Mode	IS	Mixtures	Concentration
GC-qMS (SIM)	0.1683	0.4459	<0.001
GC-TOFMS (Full scan)	0.0937	0.015	<0.001
GC-qMS (Full scan)	0.9478	0.5907	<0.001

**Table 3:** Coefficient of variation among the endogenous metabolites monitored when compared to the IS.

	GC-TOF-MS	GC-qMS	GC-SIM-MS
L-valine*	0.01	-	0.06
L-proline	0.03	0.03	0.04
L-glutamic acid	0.02	0.06	0.02
citric acid	0.02	0.02	0.02
alpha tocophereol*-		-	0.02
IS (mean)	0.04	0.05	0.06

* Endogenous metabolite missing in one or more dataset.

## Abbreviations:

AUC: Area Under the Curve
EIC: Extracted Ion Chromatogram
TIC: Total Ion Chromatogram
TMS: Trimethylsilyl
GC-MS: Gas Chromatography Coupled with Mass Spectrometry
GC-qMS: Gas Chromatography Coupled with Single Quadrupole Mass Spectrometer
GC-TOFMS: Gas Chromatography Coupled with Time of Flight Mass Spectrometer
SIM: Selected Ion Monitoring
RI: Retention Index
IS: Internal Standard
NMR: Nuclear Magnetic Resonance Spectroscopy
